# Metamaterial-Engineered Silicon Beam Splitter Fabricated with Deep UV Immersion Lithography

**DOI:** 10.3390/nano11112949

**Published:** 2021-11-03

**Authors:** Vladyslav Vakarin, Daniele Melati, Thi Thuy Duong Dinh, Xavier Le Roux, Warren Kut King Kan, Cécilia Dupré, Bertrand Szelag, Stéphane Monfray, Frédéric Boeuf, Pavel Cheben, Eric Cassan, Delphine Marris-Morini, Laurent Vivien, Carlos Alberto Alonso-Ramos

**Affiliations:** 1 Centre de Nanosciences et de Nanotechnologies, CNRS, Université Paris-Saclay, 91120 Palaiseau, France; vladyslav.vakarin@3-5lab.fr (V.V.); thi-thuy-duong.dinh@c2n.upsaclay.fr (T.T.D.D.); xavier.leroux@c2n.upsaclay.fr (X.L.R.); eric.cassan@universite-paris-saclay.fr (E.C.); delphine.morini@universite-paris-saclay.fr (D.M.-M.); laurent.vivien@c2n.upsaclay.fr (L.V.); carlos.ramos@c2n.upsaclay.fr (C.A.A.-R.); 2 LETI, University Grenoble Alpes and CEA, 38054 Grenoble, France; warren.kutkingkan@cea.fr (W.K.K.K.); cecilia.dupre@cea.fr (C.D.); bertrand.szelag@cea.fr (B.S.); 3 STMicroelectronics SAS, 850 rue Jean Monnet, 38920 Crolles, France; stephane.monfray@st.com (S.M.); frederic.boeuf@st.com (F.B.); 4 National Research Council Canada, 1200 Montreal Road, Ottawa, ON K1A 0R6, Canada; pavel.cheben@nrc-cnrc.gc.ca; 5 Center for Research in Photonics, University of Ottawa, Ottawa, ON K1N 6N5, Canada

**Keywords:** subwavelength grating, metamaterial, silicon photonics, multi-mode interference coupler, beam splitter

## Abstract

Subwavelength grating (SWG) metamaterials have garnered a great interest for their singular capability to shape the material properties and the propagation of light, allowing the realization of devices with unprecedented performance. However, practical SWG implementations are limited by fabrication constraints, such as minimum feature size, that restrict the available design space or compromise compatibility with high-volume fabrication technologies. Indeed, most successful SWG realizations so far relied on electron-beam lithographic techniques, compromising the scalability of the approach. Here, we report the experimental demonstration of an SWG metamaterial engineered beam splitter fabricated with deep-ultraviolet immersion lithography in a 300-mm silicon-on-insulator technology. The metamaterial beam splitter exhibits high performance over a measured bandwidth exceeding 186 nm centered at 1550 nm. These results open a new route for the development of scalable silicon photonic circuits exploiting flexible metamaterial engineering.

## 1. Introduction

Subwavelength grating (SWG) metamaterials consist of periodic arrangements of dielectric structures with a period substantially smaller than the wavelength of the propagating light. Within this regime, the grating effectively acts as a homogeneous material whose optical properties (e.g., effective index, dispersion, and anisotropy) are determined by the ensemble of the constituent materials and can be varied by properly designing the geometry of the grating unit cells [1,2]. This type of metamaterials have been successfully implemented in particular in silicon photonic waveguides, allowing an unprecedented control over the field distribution and propagation properties of the guided modes, largely increasing design flexibility compared to conventional waveguides [3,4,5]. SWG metamaterials can be directly integrated within established silicon-on-insulator (SOI) platforms since their fabrication uses the same process of conventional waveguides. This ease of integration fueled a large research interest and a widespread application in integrated optics. Since their first demonstration [6,7,8], a large number of devices with improved performance have been proposed, including edge couplers [9,10], surface gratings [11,12], resonators [13], filters [14], surface emitting lasers [15], directional couplers [16,17], polarization splitters, [18,19], and multi-mode interference (MMIs) couplers [20]. The use of a graded index SWG metamaterial has also been recently proposed in a III-V platform to reduce facet reflectivity [21].

The use of comparatively small grating periods represents the main technological challenge in the realization of high-performing devices based on SWG metamaterials. Structures sometimes include small features near the resolution limit of dry deep-ultraviolet (DUV) lithography tools [22]. Several demonstrations of SWG-based devices with features larger than about 120 nm and compatible with dry DUV lithography have been proposed in the literature but this normally constraints the available design space and the range of achievable material properties, making the design more complex and ultimately impacting performance [23]. For this reason, most of the successful demonstrations have so far relied on electron-beam lithography that offers higher resolution at the expense of a largely reduced throughput which limits its applicability to research or small volume productions.

In order to overcome these limitations, immersion DUV lithography has been increasingly investigated for the fabrication of photonic devices. Immersion DUV lithography is compatible with high-volume production and, compared to dry lithography, allows to achieve a three-fold improvement in device size reproducibility, with one-sigma variations below 1% across the wafer, and an almost two times reduction of line edge roughness [24,25]. These advantages result in a better on-wafer uniformity of the device performance, reduced scattering, and lower phase errors. In addition, immersion lithography has sufficient resolution to pattern small feature sizes close to 60 nm, half of what is commonly allowed by dry lithography. The significant quality improvements of immersion DUV lithography [26] allowed the demonstration of waveguides with propagation losses as low as 0.4 dB/cm [25,27], high-Q photonic crystal cavities [28], and improved across-wafer stability of ring resonators [29]. The possibility to realize sharper features has also been exploited to demonstrate highly efficient SWG edge couplers with coupling losses of 0.7 dB between the TE modes of a standard optical fiber and an integrated SOI waveguide [10]. On the other hand, the potentialities offered by immersion lithography for the realization of SWG metamaterials are still vastly unexplored, especially regarding the fabrication of photonic integrated devices with high performance and small feature sizes that would previously be accessible only by electron beam lithography.

Here, we exploit a fabrication technology based on 300-mm SOI wafers and immersion DUV lithography to experimentally demonstrate a broadband integrated beam splitter based on an SWG-engineered multi-mode interference (MMI) coupler. The device has a silicon thickness of 300 nm and nominal minimum feature size of 75 nm, well below the resolution capabilities of dry DUV lithography. Full three-dimensional finite-difference time-domain (3D FDTD) simulations show excess losses smaller than 1 dB within a broad bandwidth of 230 nm, with negligible power imbalance and phase errors. The fabricated device has a behavior well in line with simulation predictions, exhibiting high performance over a bandwidth exceeding 186 nm.

## 2. Working Principle and Device Design

**Figure 1:**
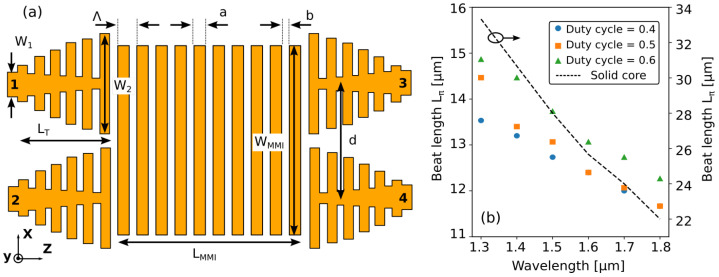
Broadband 2 × 2 MMI coupler with SWG metamaterial. (**a**) Schematic of the device. Adiabatic transitions are used to connect conventional waveguides and the MMI. (**b**) 2D FDTD simulation of the beat length L${}_{\pi }$ as a function of wavelength for W${}_{\mathrm{MMI}}$ = 3.25 µm, grating period Λ = 150 nm, and three different values of the duty cycle. As a comparison, the beat length for an MMI of the same width but based on a conventional solid silicon core instead of an SWG metamaterial core is reported with a black dashed line.

MMI couplers consist of a large waveguide section that can sustain the propagation of multiple guided modes. When light is injected in the device through one of the input ports, it excites a linear combination of these modes, each one propagating with its own propagation constant $\beta_{i}$. Interference between the excited modes generates N-fold replicas of the input excitation field at periodic intervals along the propagation direction in the multi-mode section depending on the relative phase delays between the modes (self-imaging principle [30]). If output ports are placed at the positions of the generated images, power splitting (or coupling, for reciprocity) can be achieved. For a 2 × 2 MMI coupler, such as that schematically represented in Figure 1a, the first 2-fold image of either of the two input ports is formed at a distance L = 3/2 $L_{\pi }$ (in the case of general interference [31]). $L_{\pi }$ is the beat length of the two lowest order modes of the multi-mode section

(1) $$L_{\pi }\left(\lambda \right)=\frac{\pi }{\beta_{0}\left(\lambda \right)-\beta_{1}\left(\lambda \right)},$$

with λ the wavelength of light. Because of the dispersion of the propagation constants, $L_{\pi }$ is wavelength-dependent which, in turn, causes the optimal MMI length to vary with wavelength since input replicas are generated at different positions. Since the MMI length is fixed for a given device, wavelength variations of the beat length are observed as a reduced operational bandwidth of the device. In particular, bandwidth is typically limited to about 100 nm to ensure an insertion loss penalty smaller than 1 dB in 2 × 2 MMIs with solid silicon cores [20].

In [20,32], the use of an SWG metamaterial was proposed to address this limitation. The inherit anisotropy of the SWG allows to engineer the effective material index of the multi-mode section, controlling its dispersion properties. In particular, reducing the wavelength dependence of the difference between the propagation constants of the two lowest order modes $\beta_{0}\left(\lambda \right)-\beta_{1}\left(\lambda \right)$, it is possible to mitigate the dispersion of $L_{\pi }$ and hence increase the MMI bandwidth. This approach was used here to design the device shown in Figure 1a. We consider an SOI platform with silicon core thickness of 300 nm, 2 µm buried oxide (BOX), and 2 µm upper cladding. The design was done for the transverse electric (TE) polarization. In order to operate below the Bragg condition and avoid the opening of a bandgap, the period Λ of the SWG needs to be smaller than 230 nm to ensure Λ < λ/(2n${}_{\mathrm{eff}}$) for λ> 1300 nm. Here, n${}_{\mathrm{eff}}$ is the effective index of the fundamental Floquet–Bloch mode of the grating that was approximately estimated using elementary effective permittivity theory as n${}_{\mathrm{eff}}^{2}$$≃n_{\mathrm{Si}}^{2}\cdot \mathrm{DC}+n_{{\mathrm{SiO}}_{2}}^{2}\cdot \left(1-\mathrm{DC}\right)$, assuming a duty cycle of the SWG DC = a/Λ = 0.6 [33]. The period is finally chosen to be Λ = 150 nm, well below the identified limit. Regarding the duty cycle, this is constrained between 0.4 and 0.6 to avoid feature sizes below 60 nm which is the limit for the fabrication technology.

Figure 1b shows the beat length $L_{\pi }$ as a function of the wavelength for an MMI width W${}_{\mathrm{MMI}}$ = 3.25 µm and DC = 0.4, 0.5, 0.6 when a TE mode is used as input. As a comparison, the figure shows also $L_{\pi }$ for an MMI on the same SOI platform and with the same width W${}_{\mathrm{MMI}}$ = 3.25 µm but using a conventional solid silicon waveguide core instead of the SWG metamaterial core. In order to analyze the broadband behavior of the device, beat lengths were computed over a wavelength range spanning from λ = 1300 nm to λ = 1800 nm using 2D FDTD simulations performed with the commercial software package from Ansys/Lumerical. The effective index method was applied in the vertical y-direction and material dispersion was included in the simulation. These approximated 2D simulations are in good agreement with full 3D FDTD simulation results presented in Section 3. As can be seen, for the solid core MMI, the beat length has a strong wavelength dependence and varies between 33 µm and 22 µm in the considered wavelength range. On the contrary, when the SWG core is used, $L_{\pi }$ shows a much weaker dependence on the wavelength for all the three considered duty cycles, with variations smaller than 3 µm from λ = 1300 nm to λ = 1800 nm. Moreover, $L_{\pi }$ is about half of that of the solid core case, resulting in correspondingly shorter devices. In order to maximize the minimum feature size and facilitate fabrication, we thus chose DC = 0.5 (corresponding to $L_{\pi }$ = 12.7 µm at λ = 1550 nm), resulting in a = 75 nm and b = 75 nm. The predicted optimal MMI length is L${}_{\mathrm{MMI}}≃$ 19 µm, corresponding to 127 periods of the SWG metamaterials.

The width of the access waveguides to the multi-mode section is set to W${}_{2}$ = 1.7 µm in order to ensure that only a small number of lower-order guided modes are excited, improving imaging quality [20]. The 0.4-µm-wide interconnecting silicon wire waveguides are first widened to W${}_{1}$ = 1 µm and, then, adiabatic transitions (Figure 1a) are used between these solid core waveguides and the SWG access waveguides [7]. Beside gradually adjusting the waveguide width, the transitions also adapt the core refractive index of the wire waveguide to the effective refractive index of the SWG metamaterial used in the multi-mode section in order to prevent spurious reflections at the input and output ports. Effective index matching is achieved by gradually reducing the width of the waveguide core between the SWG segments from 1 µm to 80 nm. The period and duty cycle of the SWG used in the transitions are identical to those of the multi-mode section (Λ = 150 nm, DC = 0.5). Transition losses were found to be negligible for a taper length of L${}_{T}$ = 30 µm. The distance d = 2.17 µm between the access ports was chosen to mimimize power coupling between them.

## 3. Fabrication and Experimental Results

**Figure 2:**
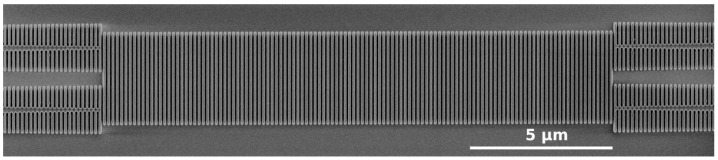
SEM image of the fabricated MMIs prior to deposition of the upper cladding.

Asymmetric Mach–Zehnder interferometers were fabricated to experimentally characterize the designed device. A range of MMI lengths from L${}_{\mathrm{MMI}}$ = 16 µm (105 SWG periods) to L${}_{\mathrm{MMI}}$ = 22 µm (145 SWG periods) was included on the mask. The devices were defined with DUV immersion lithography and transferred to the silicon layer with reactive ion etching. The SiO${}_{2}$ upper cladding was deposited with plasma-enhanced chemical vapor deposition. Figure 2 shows a scanning microscope image (SEM) of one of the MMIs before deposition of the SiO${}_{2}$ upper cladding. Feature definition is in good agreement with design specifications, with a fabricated duty cycle of 0.59.

**Figure 3:**
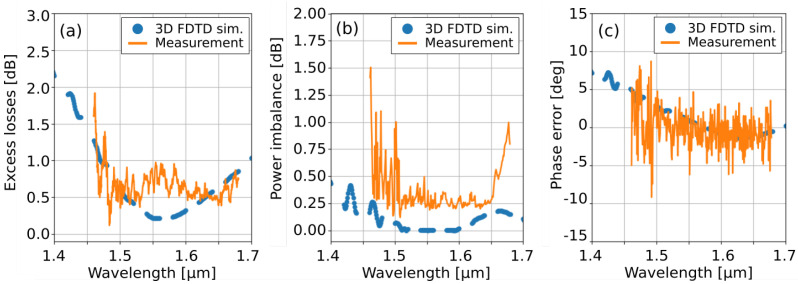
Measurement (orange solid lines) and 3D FDTD simulation (blue dashed lines) of the performance of the broadband 2 × 2 MMI, including (**a**) excess losses, (**b**) power imbalance, and (**c**) phase error. The fabricated MMI shows high performance over a 186-nm bandwidth, with losses and imbalance smaller than 1 dB and phase error smaller than 6°.

We found that with this duty cycle, MMIs with L${}_{\mathrm{MMI}}$ = 18 µm (120 periods) yielded the best performance for TE polarized light. This was assessed through full 3D FDTD simulations which included both the multi-mode section and the adiabatic transitions. In particular, we injected the fundamental TE waveguide mode at the input port 1 and simulated the transmission to the output ports 3 and 4, as marked in Figure 1a. The complex scattering coefficients $s_{31}$ and $s_{41}$ were then computed projecting the obtained field distributions at the output ports on the fundamental mode of the solid-core waveguides with W${}_{1}$ = 1 µm. These scattering coefficients were finally used to evaluate the device excess losses $10\log(|s_{31}{|}^{2}+|s_{41}{|}^{2})$, power imbalance $10\log(|s_{31}{|}^{2}/|s_{41}{|}^{2})$, and phase error $∠(s_{31}/s_{41})-\pi /2$, the same quantities measured during the experimental characterization of the device reported below. The simulation results are shown in Figure 3a–c with blue dashed lines. The MMI shows high performance near the central wavelength of λ = 1550 nm, with excess losses smaller than 1 dB from λ = 1470 nm to λ = 1700 nm. Power imbalance remains below 0.5 dB over the entire simulated wavelength range from λ = 1400 nm to λ = 1700 nm and phase errors smaller than 5° are obtained for λ> 1460 nm.

The fabricated devices have been characterized in the spectral domain using two fiber-coupled tunable laser sources covering together the 1460 nm–1680 nm wavelength range. Before entering the chip, light polarization was set to TE by means of an in-line polarization controller. On the chip, input and output grating couplers were used to couple light between integrated waveguides and standard single mode fibers. Different spectral regions were characterized by adjusting the angle of the optical fibers between 11° and 30°. The fiber collecting light at the output of the chip was finally connected to an external photodetector to measure the wavelength-dependent transmission of the device. Experimental results for L${}_{\mathrm{MMI}}$ = 18 µm are shown in Figure 3a–c with orange solid lines. Excess losses (Figure 3a), power imbalance (Figure 3b) and phase error (Figure 3c) were determined using the asymmetric Mach–Zehnder interferometers and a reference waveguide for transmission normalization [34,35,36]. The experimental results are in good agreement with 3D FDTD simulations and confirm high device performance. At λ = 1550 nm, excess losses are slightly higher than the 0.2 dB expected from simulations, which, however, do not account for the Mach–Zehnder interferometer used for the characterization. Similarly to the 3D FDTD results, the excess losses of the MMI remain smaller than 1 dB for λ > 1479 nm. Likewise, compared to simulations, fabrication imperfections only cause a minimal deterioration of 0.25 dB to power imbalance at λ = 1550 nm even if a stronger degradation occurs beyond the 1500 nm–1650 nm range, especially at shorter wavelengths. This is likely due to the reduced transmission efficiency of the input and output grating couplers for λ < 1500 nm which complicates the accurate estimation of the MMI power imbalance from the extinction ratio of the Mach–Zehnder interferometer. This can also be noticed in the phase error measurements, which show an increased noise for λ < 1500 nm. Despite this, phase errors smaller than ±6° are obtained for λ > 1494 nm. Our sources do not extend beyond λ = 1680 nm, preventing measurements at longer wavelengths. Aiming for excess losses and imbalance below 1 dB and phase error smaller than 6°, these results yield an experimentally measured bandwidth of at least 186 nm.

## 4. Discussion and Conclusions

We have reported on the use of a 300-mm SOI fabrication platform with DUV immersion lithography to implement a high-performing MMI beam splitter based on an SWG metamaterial. The high resolution of immersion lithography allowed designs with nominal feature size of 75 nm. The use of an SWG core instead of a conventional solid silicon core resulted in a broadband and compact MMI, with about a two times reduction in the length of the multi-mode section. The device size could be further scaled down by reducing the length of the input and output tapers, which represent the longest sections in the current design.

The experimental characterization of devices showed low excess losses below 1 dB with negligible power imbalance and phase errors over a bandwidth of 186 nm, confirming the high quality of the fabrication and making the device suitable, for example, for coarse wavelength division multiplexing transceivers. These results demonstrate for the first time SWG metamaterials with a fabrication process compatible with high-volume production while simultaneously reaching feature sizes that would commonly require electron-beam lithography. We believe that the use of lithographic techniques routinely available in complementary metal-oxide-semiconductor (CMOS) processes will be of fundamental importance to bring the potentialities of refractive index engineering toward commercial exploitation. This will enable the fabrication of high-performance devices for fiber-to-chip coupling, power splitting, polarization management, and spectral filtering, with promising applications, for example, in coherent communications, sensing, and spectroscopy.

## Data Availability

The data presented in this study are available from the corresponding author upon request.
